# Epidemiology of inherited metabolic disorders in newborn screening: insights from three years of experience in Southern Iran

**DOI:** 10.1186/s13023-025-03602-w

**Published:** 2025-02-25

**Authors:** Leila Salarian, Homa Ilkhanipoor, Anis Amirhakimi, Zhila Afshar, Saman Nahid, Fariba Moradi Ardekani, Nazila Rahimi, Negar Yazdani, Abdolhossein Nikravesh, Zahra Beyzaei, Hossein Moravej

**Affiliations:** 1 Neonatal Research Center, Shiraz University of Medical Sciences, Shiraz, Iran; 2 Department of Pediatric Endocrinology and Metabolism, School of Medicine, Shiraz University of Medical Sciences, Shiraz, Iran; 3 Department of Biochemical Genetics, Saman Lab, Shiraz, Iran; 4 Head of Non-Communicable Diseases Group, Shiraz University of Medical Sciences, Shiraz, Iran; 5 Public Health expert Non-Communicable Diseases Group, Shiraz University of Medical Sciences, Shiraz, Iran; 6 Department of Nursing, Community based Psychiatric Care Research Center, School of Nursing and Midwifery, Shiraz University of Medical Sciences, Shiraz, Iran; 7 Department of Pediatric Endocrinology & Metabolism, Bushehr University of Medical Sciences, Bushehr, Iran; 8 Shiraz Transplant Research Center (STRC), Shiraz University of Medical Sciences, Shiraz, Iran

**Keywords:** Metabolism, Inborn errors of metabolism, Neonatal screening, Genetic diseases, Inherited metabolic disease, Dried blood spots

## Abstract

**Background:**

Newborn screening is essential for the early detection of congenital genetic and metabolic disorders, enabling timely intervention to prevent morbidity, mortality, and disabilities associated with inherited metabolic disorders (IMDs). The Iranian Neonatal Screening Program piloted in Fars Province, screening nearly 100% of neonates for 20 disorders. This study aimed to assess the epidemiology of these metabolic diseases. From March 2019 to September 2021, 138,689 neonates were screened using tandem mass spectrometry (MS/MS) on dried blood spots. Those with abnormal results were referred to pediatric endocrinology and metabolism specialists for confirmatory testing per American College of Medical Genetics guidelines.

**Results:**

Among the screened neonates, 139 patients of IMDs were identified, yielding an estimated birth prevalence of 1:1000. The positive cases included 55 aminoacidopathies, 47 organic acidemias, 31 fatty acid oxidation disorders, and 6 urea cycle defects were detected. The most prevalent IMDs were phenylalanine metabolism disorders, short-chain acyl-CoA dehydrogenase deficiency, 3-methylcrotonyl-CoA carboxylase deficiency, and methylmalonic acidemia. Notably, the prevalence of IMDs in Fars Province is significantly higher than average global statistics. Additionally, we observed that certain disorders previously deemed very rare exhibit a relatively high prevalence in this region.

**Conclusions:**

Our data highlight the efficiency and robustness of neonatal screening for IMD in Iran. It demonstrates the need for expanded screening efforts across the entire country. One limitation of this study is that the screening was conducted in only one state, which may not reflect the broader population of Iran. Future research should involve nationwide implementation of screening programs to validate our findings and assess the prevalence of IMDs in diverse regions. Furthermore, exploring the applicability of our screening methods in other Middle Eastern countries could help promote early and life-changing diagnoses across the region.

## Introduction

Inherited metabolic disorders (IMDs) are a complex group of genetic disorders caused by defective enzymes, cofactors, or transporters engaged in metabolic pathways [1]. The incidence of these disorders is individually rare (< 1:100,000) but considering the diversity of the enzymatic pathways, the collective incidence is high, around 1:800 to 1:2500 live births [2].

Numerous IMDs can lead to severe clinical consequences including irreversible mental retardation, coma, physical handicaps, lethargy, and even death among neonates. A significant percentage of inborn errors of metabolism (IEMs) are curable. Therefore, timely and precise screening is essential to prevent or reduce the morbidity and mortality of these disorders before the onset of symptoms [3, 4].

In many countries, neonatal screening is a routine program for congenital hypothyroidism (CH) and phenylketonuria (PKU). However, the panel of disorders screened varies widely in developed countries. For example, 17 metabolic disorders in Netherlands, 13 in Denmark, and 12 in Germany are being screened. In the United States, nearly all of 4.3 million newborns are screened for more than 30 metabolic conditions. The United Kingdom (UK) screening programs is only for PKU and Medium-chain acyl-CoA dehydrogenase (MCAD) deficiency [5–7].

In our country, newborn screening has been mandatory since 2002 for congenital hypothyroidism (CH), phenylketonuria (PKU), and glucose-6-phosphate dehydrogenase (G6PD) deficiency. While the high rate of consanguineous marriages in Iran (approximately 38.6% with a mean inbreeding coefficient of 0.018) contributes to an increased incidence of autosomal recessive diseases, other factors also underscore the importance of comprehensive neonatal screening. Advances in diagnostic technologies, the growing recognition of the public health burden of inborn errors of metabolism (IEMs), and the cost-effectiveness of early detection further justify the need for expanded screening programs. In response, neonatal screening for 20 IEMs was launched in Fars Province in March 2019. This study reports the results of these screening tests conducted from March 2019 to September 2021, highlighting the prevalence of IEMs and the importance of implementing tailored screening strategies to address the genetic and public health challenges in this region.

## Methods

This cross-sectional descriptive study was conducted from March 2019 to September 2021 in Fars province, Iran. All neonates living in Fars or born in the hospitals affiliated to Shiraz University of Medical Sciences who had undergone neonatal screening for IMDs were enrolled in the study. Newborns with abnormal metabolic screening results—defined as biochemical markers exceeding established cutoff values indicative of potential IEMs—were included only if they had a confirmed final diagnosis through follow-up diagnostic tests. Newborns with screening results that could not be confirmed due to inconclusive diagnostic outcomes were excluded. Additionally, Infants with incomplete chart documentation were excluded.

In Fars province, all neonates are screened for 20 inherited metabolic diseases three to five days after birth. These disorders were selected based on their clinical significance, prevalence in the population, their treatability with medication, and the availability of cost-effective diagnostic and treatment options. The metabolic screening test is performed using Tandem Mass Spectrometry (MS/MS) method in the reference laboratory. This test measures normal cut-off value of specific amino acids and acylcarnitines tested in dried blood spots (DBS) obtained from heel prick. The metabolites measured include: Phenylalanine, Tyrosine, Glycine, Alanine, Valine, Leucine & Isoleucine, Arginine, Citrulline, Methionine, Ornithine, C0, C2, C3, C3DC + C8OH, C4, C4OH, C4DC, C5, C5:1, C5OH, C5DC + C10OH, C6, C6OH, C6DC, C8, C8:1, C10, C10:1, C10:2, C12, C12:1, C14, C14:1, C14:2, C14OH, C16, C16:1, C16OH, C16:1OH, C18, C18:1, C18:2, C18OH, and C18:1OH.

The selection of the 20 metabolic disorders for screening is based on their prevalence in the region and the potential for early intervention to improve health outcomes. The metabolic screening is designed to detect diseases such as Argininosuccinic Aciduria, citrullinemia type 1, Maple Syrup Urine Disease, Homocystinuria, Phenylketonuria classic, Tyrosinemia Type 1, Primary Carnitine Deficiency, Medium Chain Acyl-CoA Dehydrogenase Deficiency, Very Long Chain Acyl-CoA Dehydrogenase Deficiency, Long Chain 3-Hydroxyacyl-CoA Dehydrogenase Deficiency, Trifunctional Protein Deficiency, Propionic Acidemia, Methylmalonic Acidemia, methyl malonyl-CoA mutase, Methylmalonic Acidemia: Cobalamin Disorders, Isovaleric Acidemia, 3-methylcrotonyl-CoA Carboxylase deficiency, 3-Hydroxy-3-Methylglutaric Aciduria, Holocarboxylase Synthetase deficiency, B-Ketothiolase Deficiency, and Glutaric Acidemia Type-1. The targeted metabolic disorders in newborn screening in Iran is shown in Table 1.

**Table 1:** Targeted metabolic diseases included in newborn screening programm in Iran

20 Core conditions	26 Secondary conditions
Argininosuccinic Aciduria	Argininemia
Citrullinemia Type 1	Citrullinemia Type 2
Maple syrup urine disease	Hypermethioninemia
Homocystinuria	Benign Hyperphenylalaninemia
Phenylketonuria classic	Biopterin biosynthesis/regeneration defect
Tyrosinemia Type 1	Non-Ketotic Hyperglycinemia
Primary carnitine deficiency/carnitine transporter defect	Ornithine Transcarbamylase Deficiency
Medium chain Acyl-CoA dehydrogenase deficiency	Carbamoyl Phosphatase Synthetase 1 Deficiency
Very long chain Acyl-CoA Dehydrogenase Deficiency	HHH Syndrome
Long Chain 3-Hydroxyacyl-CoA Dehydrogenase Deficiency	Tyrosinemia Type 2, 3
Trifunctional Protein Deficiency	Short chain Acyl-CoA Dehydrogenase Deficiency
Propionic Acidemia	Medium/Short chain Acyl-CoA Dehydrogenase Deficiency
Methylmalonic Acidemia: methylmalonyl-CoA mutase	Glutaric Acidemia Type 2
Methylmalonic Acidemia: Cobalamin Disorders	Medium Chain ketoacyl-CoA Thiolase Deficiency
Isovaleric Acidemia	2,4-Dienoyl-CoA Reductase Deficiency
3-Methlcrotonyl-CoA Carboxylase Deficiency	Carnitine Palmitoyltransferase 1 Deficiency
3-Hydroxy-3-Methylglutaric Aciduria	Carnitine Palmitoyltransferase 2 Deficiency
Holocarboxylase Synthetase Deficiency	Carnitine Acylcarnitine Translocase Deficiency
Β-ketothiolase Deficiency	Methylmalonic Acidemia with Homocystinuria
Glutaric Acidemia Type 1	Malonic Acidemia
	Isobutyrylglycinuria
	2-Methylbutyrylglycinuria
	3-Methylglutaconic Aciduria
	2-Methyl-3-hydroxybutyric Aciduria

Before starting the screening program, the gray zone, normal range, and pathological zone for each metabolite were determined by the laboratory team based on preliminary studies conducted on the healthy population. The gray zone refers to a range where values show slight deviation from the normal range and, in some cases, may also be observed in healthy individuals. Newborns whose metabolites fall entirely within the normal range are reassured, and their parents are informed that no further investigation is necessary. Cases with one or more metabolites in the pathological range are referred to a doctor for further evaluation. Cases whose metabolites fall in the gray zone are asked to return to the laboratory for a repeat screening. If the metabolite levels remain in the gray or pathological zone, the baby will be referred to a physician.

All newborns with abnormal levels of one or more metabolites are referred to a physician with subspecialty expertise in inherited metabolic diseases. The decision-making process for referrals is guided by the severity of the abnormal results, the presence of abnormal metabolites at the same time, and established protocols. The assigned expert takes a detailed history, performs a comprehensive physical examination, and requests the necessary laboratory tests according to American College of Medical Genetics and Genomics (ACMG) guideline.

Once the requested test results are available, a diagnosis is made, and the confirmatory genetic assay are performed. This included a targeted genetic assay conducted using next-generation sequencing (NGS) to ensure accuracy and specificity. The NGS analysis focused on specific genes or regions of interest, providing high-resolution insights into genetic variations relevant to the diagnosis. Information from the screening and supplementary tests is recorded in a data collection form that includes demographic data, abnormal values according to the neonatal screening, confirmatory tests, genetic study, and the final diagnosis confirmed by the expert consultation. Infants are evaluated and categorized based on their metabolic disorders. This study was approved by the Ethics committee of Shiraz University of Medical Sciences (Ethics code: IR.sums.med.rec.1400.291). In this study, demographic and descriptive data were presented as frequencies (%) for categorical variables using SPSS 22.0 for Windows (SPSS Inc., Chicago, IL, USA).

## Results

**Fig. 1:**
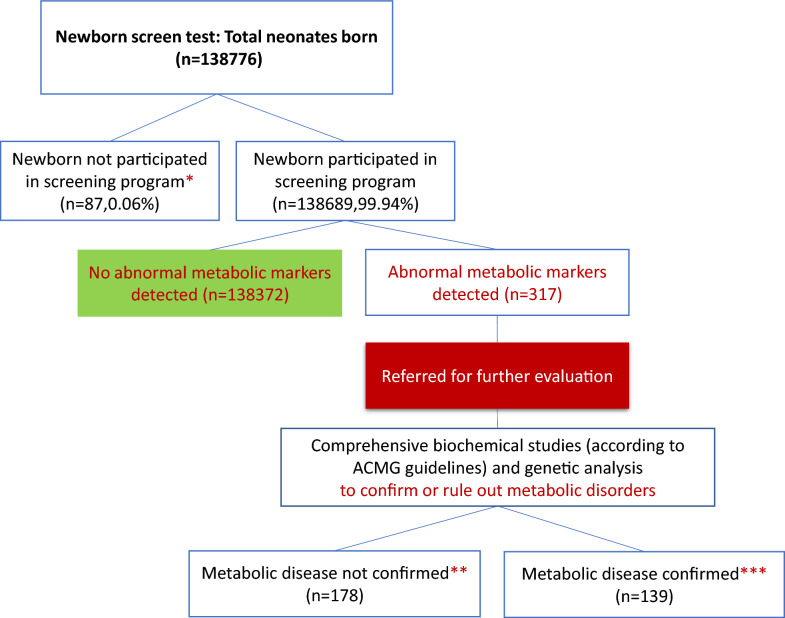
Newborn screening analytical process: This flowchart illustrates the steps in the newborn screening process for metabolic disorders. After a heel prick test within the first few days of life, blood samples are analyzed using tandem mass spectrometry. If initial results indicate potential disorders, families are contacted for a repeat test. Positive results lead to recalling patients for confirmatory testing and referral to specialists, enhancing early intervention and improving health outcomes. * Opted out of screening or not accessible for screening. ** No evidence of a metabolic disorder after confirmatory testing. *** Diagnosis of metabolic disease confirmed

In the 3-year, 6-month period between March 2019 and September 2021, a total of 138,776 neonates were born in 32 cities of Fars state in hospitals affiliated to Shiraz University of Medical Sciences. Of them 138,689 newborns (99.94%) underwent neonatal screening for IMDs by DBS. Figure 1 outlined the analytical process for newborn screening of metabolic disorders. If the initial screening suggests a disorder, families were informed, and a repeat test is conducted for confirmation. Positive results lead to further diagnostic testing and referral to specialists, facilitating early intervention and better health outcomes for affected infants. Of 138,689 newborns, 317 neonates were recalled for the second DBS sampling due to abnormal metabolic screening tests. Among these, 139 cases were diagnosed with IMDs, leading to a prevalence of IMD in Fars province of 1 in 1000 screened newborns. Of these cases, 67 (48.2%) were male and 72 (51.8%) were female. The study identified a total of 21 different IMDs, comprising 4 aminoacidopathies (39.6%, 55 cases), 10 organic acid disorders (33.8%, 47 cases), 4 fatty acid oxidation disorders (FAOD) (22.3%, 31 cases), and 3 urea cycle defects (4.3%, 6 cases).

The most prevalent IMDs identified in the study was phenylalanine metabolism disorder, which affected 42 patients (30%) with a prevalence of 1 in 3,333 live births. Among them, 13 cases of hyperphenylalaninemia (Phe 2–10), 12 cases of mild phenylketonuria (Phe 10–20), 11 cases of classic form of phenylketonuria (phe > 20), and 6 patients with non-classic form (tetrahydrobiopterin metabolism disorder) were detected. Furthermore, 58 cases of transient hyperphenylalaninemia were observed, which were not considered in the statistical analysis of the metabolic diseases (Table S1). This rate is higher than the global average of 1 in 10,000. The observed discrepancy may be attributed to factors such as the high rate of consanguineous marriages in this region, which increases the likelihood of autosomal recessive disorders like phenylalanine metabolism disorders. Furthermore, improved diagnostic capabilities and robust newborn screening programs in this area may contribute to the higher detection rates compared to global averages. Among organic acidemias, the leading disorders were 3-methylcrotonyl-CoA carboxylase deficiency (7.9%, 11 cases) and methylmalonic acidemia (7.2%, 10 cases). The prevalence of these disorders was 1 in 12,500 and 1 in 14,250, respectively. Both conditions exhibit lower global prevalence rates, estimated to be between 1 in 30,000 and 1 in 50,000 for 3-methylcrotonyl-CoA carboxylase deficiency, and between 1 in 48,000 and 1 in 61,000 for methylmalonic acidemia (Table 2). Additionally, B-ketothiolase deficiency was identified in one newborn (0.71%) as detailed in Table S1.

**Table 2:** Positive Cases Detected Through Newborn Screening (NBS) Using Tandem Mass Spectrometry (MS/MS) from 2019 to 2021, Along with Birth Prevalence Data in Iran

Classes	Result of screening	Birth prevalence	Worldwide birth prevalence*
**Aminoacidopathies**	**Total Cases**: 55 (39.65%) **Most Prevalent Disorder**: **Phenylalanine metabolism defects**, (42 cases, 30.21%)	1:2,500 1:3,333	Unknown 1:10,000
	*Notable Subtypes:* *Hyperphenylalaninemia, (13 cases, 9.35%)* *Classic PKU, (11 cases, 7.91%)*		
**Organic acid disorders**	**Total Cases**: 47 (33.81%)	1:2,525	1:20,000 to < 1:200,000
	**Leading Disorders:** *3-Methylcrotonyl CoA carboxylase deficiency, (11 cases, 7.91%)* *Methylmalonic acidemia, (10 cases, 7.20%)*	1:12,500 1:14,250	1:30,000 to 1:50,000 1:48,000 to 1:61,000
**Fatty Acid Oxidation Defects (FAOD)**	**Total Cases**: 31 (22.30%)	1:4,454	1:6,500 to 1:110,000
	**Common Disorders**: *Short-chain acyl-CoA dehydrogenase deficiency, (12 cases, 8.63%)* *Medium-chain acyl-CoA dehydrogenase deficiency, (10 cases, 7.20%)*	1:12,500 1:14,250	Unknown 1:4,900 to 1:27,000
**Urea Cycle Defects**	**Total Cases**: 6 (4.32%) **Identified Conditions**: *Citrullinemia type 1, Argininosuccinic aciduria and Carbamoylphosphate synthetase (CPS), (2 cases each)*	1:23,460	1:35,000

The second most common disorder identified through newborn screening in Iran was short-chain acyl-CoA dehydrogenase deficiency, detected in 12 patients (8.3%), although its global prevalence remains unknown. Among fatty acid oxidation defects (FAOD), the following conditions were identified in newborns: medium-chain acyl-CoA dehydrogenase deficiency in 10 patients (7.2%), carnitine uptake defect in 7 patients (5%), and glutaric acidemia type 2 in 2 patients (1.4%). For Urea cycle defects, the following conditions were detected in two newborns each (1.4%): Citrullinemia type 1, Argininosuccinic aciduria, Carbamoylphosphate synthetase (CPS) deficiency (Table 2). These prevalence rates are lower than the global averages, as shown in Table S1.

The high prevalence of IMDs in Fars province underscores the critical need for strengthening healthcare infrastructure to address the burden of these disorders. This includes expanding neonatal screening programs, increasing access to genetic counseling services, and establishing specialized centers for the treatment and long-term management of metabolic diseases to improve health outcomes and reduce the socio-economic impact on affected families.

## Discussion

In this study, 139 patients with various IMDs were diagnosed. The prevalence of these diseases in Fars province, according to newborn screening (NBS), was 1 per 1000 live births. This prevalence indicates one of the higher rates reported worldwide.

In this regard, a systematic review by Waters et al. reported a total prevalence of IMDs at birth of 50.9 per 100,000 live births. Notably, the prevalence in the Eastern Mediterranean was higher, at 75.7 per 100,000 live births, which aligns with the region's higher rates of parental consanguinity [10]. This genetic factor can significantly increase the risk of recessive genetic disorders, thereby contributing to the higher prevalence of IMDs. Moreover, a study by Zhang et al. in northwest China, identified 75 infants with IMDs, corresponding to a prevalence of 51 per 100,000 live births (1 in 1949) [11]. In contrast, a study in Denmark, Iceland, and Greenland by Lund et al. reported that among 504,049 screened infants over seven years, 114 cases were confirmed positive, yielding a frequency of 1 in 4942 [12]. The higher prevalence of IMDs in Fars province compared to these studies underscores the critical role of metabolic screening, especially in communities with elevated rates of consanguineous marriage. Factors such as the sensitivity of screening tests, regional genetic backgrounds, and healthcare access may contribute to these differences. For instance, variations in the types of disorders screened and the methodologies employed can influence prevalence rates. Additionally, cultural practices, including consanguinity, may further exacerbate the incidence of IMDs in Fars province. Overall, these comparisons highlight the importance of tailored screening programs in regions like Fars, where genetic factors may significantly affect the prevalence of metabolic disorders. Understanding these dynamics is essential for improving early detection and intervention strategies.

It is estimated that about 0.45 million people worldwide are affected by PKU. The overall global prevalence is 1:23,930 live births, and different types of the disease have different geographical distributions. The prevalence of the classic form is reported to be 56% in Europe, 71% in East Asia, and 80% in Australia [13]. In a systematic review and meta-analysis by Shokri et al. in Iran, in 18 studies involving 3,339,327 Iranian infants, the prevalence of suspected cases of hyperphenylalaninemia was 45.6 per 100,000, phenylketonuria 16.5 per 100,000, mild and moderate hyperphenylalaninemia 9.7 per 100,000, and classic PKU was equivalent to 4.4 per 100,000. The prevalence of PKU varies among different geographical subgroups of Iran [14]. The results on the prevalence of phenylalanine metabolism disorders in this study align with those previously reported in Iran.

Following phenylketonuria, the study identified two prevalent fatty acid beta-oxidation disorders— SCAD and MCAD deficiency—as among the most commonly detected IMDs through screening. These disorders follow an autosomal recessive pattern of inheritance, leading to impaired fatty acid metabolism in mitochondria. They are characterized by significant clinical, biochemical, and genetic heterogeneity [15]. The prevalence of fatty acid metabolism disorders was reported as 6.51 per 100,000 in a systematic review, with MCADD being the most common [10]. For MCADD, global prevalence estimates are around 1 in 15,000 in the U.S. and approximately 1 in 17,000 in Europe. In our study, we found a prevalence of 1 in 14,250, which aligns closely with these global figures, highlighting a comparable yet elevated prevalence in our cohort. The higher prevalence observed in our study may be influenced by both environmental and genetic factors. Geographical and regional variations in genetic mutations, such as founder effects or consanguinity rates, could contribute to this disparity. Additionally, environmental factors like dietary practices or healthcare access, which may impact the detection and management of MCADD, might play a role. Given that MCAD deficiency can cause severe cardiovascular and brain complications and even sudden death if left undiagnosed [16], the IMD screening program will play an important role in preventing these problems in the society.

SCADD is one of the most common IMDs, with a prevalence of approximately 1 in 35,000–50,000. A study by Maldegem et al. reported a prevalence of 1 in 50,000 for SCADD, suggesting that it may be more prevalent than previously believed. However, often considered clinically insignificant due to its nonspecific symptoms and typically benign course; many affected individuals remain asymptomatic and do not meet the criteria for neonatal screening [17]. In contrast, global data indicates that SCADD has a prevalence ranging from 1 in 50,000 to 1 in 100,000 in Europe. Our findings, showing a prevalence of 1 in 4,454, suggest a notably higher rate in our population. A study by Messina et al. [18], which included 50,521 infants undergoing IMDs screening, identified SCADD as the second most prevalent diagnosed disorder, with a prevalence of 1 in 8420. Among the 11 patients diagnosed with SCADD, most remained asymptomatic despite positive screening results. Understanding its prevalence in Fars province can inform healthcare providers about potential biochemical phenotypes and guide monitoring and interventions, even for those who remain asymptomatic. However, given the asymptomatic nature of SCADD, the effectiveness and cost-effectiveness of screening programs remain unclear and require further evaluation and additional data.

Countries with comparable prevalence rates have adopted various strategies to enhance the effectiveness of IMD screening programs. For instance, the United States has implemented comprehensive newborn screening protocols using tandem mass spectrometry, complemented by public awareness initiatives to improve compliance with dietary interventions for MCAD deficiency [19]. In the European Union, standardized protocols and long-term follow-up registries, such as those for fatty acid oxidation disorders, ensure continuity of care and support evidence-based management [21–24]. Additionally, targeted genetic screening programs in regions with high consanguinity rates, such as parts of China, address population-specific genetic variability [25, 26]. These international approaches provide valuable insights for optimizing IMD screening and management in our context.

The third most prevalent IMDs identified after phenylketonuria and SCAD deficiency was 3-methylcrotonyl carboxylase deficiency (3MCCD), with 11 cases reported (1 in 12,500). There are no available statistics on the prevalence of metabolic diseases in Iran that we can use to compare with our screening results. Forsyth et al. noted that 3MCCD, an organic acidemia caused by a defect in leucine metabolism, was previously considered rare but is now recognized as a common metabolic disorder diagnosed through newborn screening [27]. Symptoms can include developmental delay, hypoglycemia, growth abnormalities, and metabolic acidosis [28, 29]. Recent screening has revealed an increased prevalence of 3MCCD (1 in 36,000), although most patients present clinically normal. This suggests that the disorder may represent a biochemical phenotype rather than a full-blown disease, or it may have low genetic penetrance, with expression influenced by environmental and genetic factors. Thus, elevated levels of C5OH detected in newborn screening alone are insufficient for diagnosis or prognosis. The higher prevalence observed in our study compared to previous reports may be attributed to the high rate of consanguineous marriages in Iran and Fars province. This area has not been extensively studied, making our findings a valuable resource for future research. Notably, Moravej et al. highlighted that the prevalence of cerebral creatine deficiency in Iran exceeds that of other nations, indicating the need for national planners to reassess the screening program [30]. It may be prudent to consider incorporating additional disorders, such as cerebral creatine deficiency, into the newborn screening initiative.

These findings can provide critical insights for regions with high consanguinity, where genetic factors significantly influence IMD prevalence. Policymakers in these regions can prioritize expanding newborn screening programs, integrating genetic counseling services, and investing in public education to reduce the burden of IMDs. Furthermore, incorporating population-specific genetic data into screening protocols can improve early detection rates and guide resource allocation for preventive measures and healthcare interventions.

Several limitations of our study should be acknowledged. First, excluding neonates with incomplete chart documentation or unconfirmed diagnoses may underestimate the true prevalence of IMDs, as these cases could represent undetected or misclassified disorders. Second, neonates from less accessible areas may not be adequately represented, potentially skewing the prevalence data. Furthermore, the reliance on specific screening protocols might limit the generalizability of our findings to other regions or populations with different screening practices. Future research should focus on long-term follow-up of diagnosed cases to assess health outcomes, as well as exploring the genetic factors contributing to the high prevalence of IMDs in this region. This could provide a more comprehensive understanding of the underlying causes and aid in developing targeted interventions.

Currently, metabolic screening is performed in few provinces in Iran. After the identification and confirmation of IMDs in patients, referral patterns and access to healthcare services are established. Patients are followed up by an IMDs center, where they receive necessary supports such as specialized formula, medications, and dietary guidance. Patients are seen by a multidisciplinary team, including subspecialist physicians, nutritionists, psychologists, and nurses, all of whom are experienced in the management of IMDs. Patients are referred to other specialties or rehabilitation centers as needed. Our screening program is intended to expand nationwide in the future. Given the high rates of consanguineous marriage across the Middle East, it would also be prudent to extend screening for metabolic disorders to neighboring countries. Such actions could significantly improve early detection and intervention, ultimately enhancing public health outcomes in the region. Iran’s comprehensive screening program serves as a model, while neighboring countries like Iraq primarily screen for only a few conditions [31]. By sharing best practices, Iran can assist its neighbors in improving screening capabilities. Additionally, discussions on the cost-effectiveness of broader screening can address resource allocation concerns, demonstrating that investment in comprehensive screening yields significant long-term health benefits. This comparison emphasizes Iran’s advancements and the potential for regional collaboration in public health.

## Conclusion

To conclude, expanding newborn screening for IMDs across Iran and the Middle East is vital, as the findings from Fars province reveal a significantly higher prevalence than global averages. Early detection has proven to improve patient outcomes by reducing morbidity and mortality, emphasizing the need for comprehensive screening programs nationwide and in neighboring countries. The most prevalent disorders identified—PKU, SCADD, MCADD, and 3MCCD—highlight the urgency of implementing this pilot screening initiative throughout the region.

To achieve this, a phased expansion of the screening program should be prioritized, starting with high-prevalence regions and gradually scaling up to national coverage. Strengthening laboratory infrastructure, training healthcare providers, and establishing regional referral centers are critical first steps. Additionally, increasing public and stakeholder awareness through targeted education campaigns can foster community support and ensure program sustainability. Re-evaluating and broadening the screening panel to include other critical disorders will further enhance public health strategies. Furthermore, long-term follow-up studies are crucial to measure the outcomes of early diagnosis and treatment, ensuring that the benefits of screening translate into improved health and quality of life for affected individuals. Drawing lessons from the experiences of other Middle Eastern or low-resource countries can reveal cost-effective approaches and address implementation challenges. Collaborations with global health organizations and sharing best practices regionally can provide valuable insights and resources to optimize outcomes. By taking these decisive actions, we can protect the health of future generations, reduce the societal and economic burden of IMDs, and contribute to broader public health advancements across the region.

## Data Availability

The datasets analyzed during this study are not publicly available due to privacy and ethical restrictions but are available from the corresponding author on reasonable request.

## Abbreviations

CPS: Carbamoylphosphate synthetase
CH: Congenital hypothyroidism
FAOD: Fatty acid oxidation defects
G6PD: Glucose-6-phosphate dehydrogenase
IEM: Inborn errors of metabolism
IMD: Inherited metabolic disease
MCAD: Medium-chain acyl-coenzyme A dehydrogenase
NAGS: N-acetyl glutamate synthetase
NBS: Newborn screening
PKU: Phenylketonuria
SCAD: Short chain acyl-CoA dehydrogenase deficiency
